# Novel pyridine-based Pd(II)-complex for efficient Suzuki coupling of aryl halides under microwaves irradiation in water

**DOI:** 10.1186/s13065-017-0320-2

**Published:** 2017-09-18

**Authors:** Ismail I. Althagafi, Mohamed R. Shaaban, Aisha Y. Al-dawood, Ahmad M. Farag

**Affiliations:** 1 Department of Chemistry, Faculty of Applied Science, Umm Al-Qura University, Makkah Almukaramah, Mecca, Saudi Arabia; 2 Department of Chemistry, Faculty of Science, Cairo University, Giza, 12613 Egypt

**Keywords:** Palladium precatalyst, Suzuki–Miyaura, C–C cross-coupling, Microwave irradiation

## Abstract

Suzuki C–C cross-coupling of aryl halides with aryl boronic acids using new phosphene-free palladium complexes as precatalysts was investigated. A pyridine-based Pd(II)-complex was used in open air under thermal as well as microwave irradiation conditions using water as an eco-friendly green solvent.

## Introduction

**Fig. 1:**
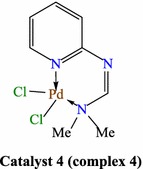
Pyridylformamidine-based Pd(II)-complexe **4** (catalyst **4**)

Palladium is a versatile metal for homogeneous and heterogeneous catalyses [1–4]. Homogeneous palladium catalysis has gained enormous relevance in various coupling reactions, especially in Suzuki reaction. Many products could be synthesized by this methodology for the first time, or in a much more efficient way than before. This kind of catalysis provides high reaction rate and high turnover numbers (TON) and often affords high selectivity and yields [5–7]. Control and use of such Pd catalysts can be tuned by ligands, such as phosphines, amines, carbenes, dibenzylideneacetone (dba), etc. Proper ligand construction has led to catalysts that tolerate weak leaving group such as chloride, exhibit higher TON and reaction rates, improved lifetimes, and are stable to run the reactions without the exclusion of water or air and at lower temperatures [8, 9]. Recently, there has been considerable interest in the designing of novel phosphorus-free palladium catalysts for higher activity, stability and substrate tolerance that allow reactions to be carried out under milder reaction conditions [10, 11]. Formamidines are of high interest in synthetic chemistry [12, 13] and have been used extensively as pesticides [14–18] and as pharmacological agents [19–21]. They are versatile ligands, capable of forming flexible coordination modes which lead to various molecular arrangements [22, 23]. Transition metal complexes of formamidinates display novel electronic properties and recently show an extraordinary ability to stabilize high oxidation states [24–30]. On the other hand, reactions that can proceed well in water, which has been reported to be a powerful green solvent, because of its safe and environmentally benign properties [31]. Also, microwave irradiation methodology received a growing interest as a heating source, because of its achievements in green organic synthesis [32–34]. In continuation of our research work concerned with the use of Pd(II)-complexes in C–C cross coupling reactions in water, under thermal heating as well as microwave irradiation conditions, [35, 36] we report here our study on the catalytic activity of the hitherto unreported, easily accessible *N*,*N*-dimethyl-*N’*-pyridyl formamidine-based Pd(II)-complex **4** (catalyst **4**) (Fig. 1) as a precatalyst in the Suzuki cross-coupling of aryl halides with a variety of arylboronic acids, in water, under thermal heating as well as microwave irradiation conditions.

## Results and discussion

### Preparation of the Pd(II)-complex **4** (catalyst **4**)

**Scheme 1:**
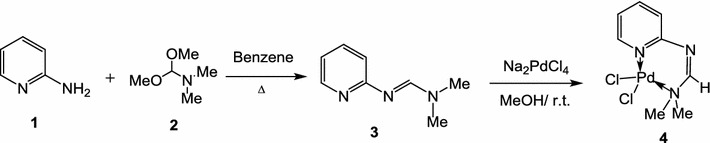
Preparation of the Pd-complex **4** (catalyst **4**)

2-Aminopyridine (**1**) was treated with dimethylformamide dimethyl acetal (**2**), in benzene, to afford the formamidine derivative **3** as shown in Scheme 1. The Pd(II)-complex **4** was prepared by dissolving the formamidine derivative **3** in methanol followed by addition of an equimolar amount of sodium tetrachloropalladate, in methanol, at room temperature (Scheme 1). The structure of complex **4** was established based on its elemental analyses and spectroscopic data. The ^1^H NMR spectrum of the complex **4** showed a singlet signal at δ 3.56 due to *N*,*N*-dimethylamino protons, in addition to a multiplet at δ 6.53–6.55, two doublet signals in the region at δ 7.26–7.48 due to pyridine ring protons and a singlet at 8.35 due to the formamidine proton. The chemical shift of the protons of the two methyl groups of *N*,*N*-dimethylamino group indicates the effect of the coordination of the nitrogen atom of the *N*,*N*-dimethylamino group with the Pd metal. Comparison of the chemical shift of the same protons in the metal free ligand showed that the resonance of the protons at more down field of the spectrum due to the strong electropositive nature of the metal ion. The IR spectrum of the complex **4** showed a characteristic band at 1629 cm^−1^ due to the C=N function and a band at 771 cm^−1^ due to the Pd–N bond vibration.

### Suzuki cross-coupling reactions of aryl bromides

Factors affecting the optimization of the catalytic activity of complex **4** in Suzuki cross-coupling reactions are given in the following sections.

#### Effect of concentration of the catalyst **4** on the coupling of *p*-bromoacetophenone with phenylboronic acid in water

**Scheme 2:**
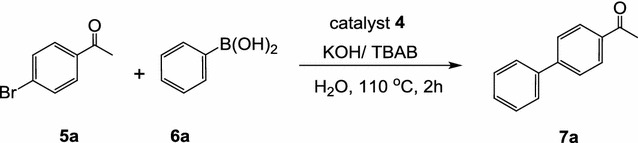
Effect of concentration of catalyst **4** on the coupling of p-bromoacetophenone with phenylboronic acid

Effect of concentration of the catalyst **4** on the cross-coupling reaction of phenylboronic acid with *p*-bromoacetophenone, in water using potassium hydroxide and tetrabutylammonium bromide (TBAB) as a co-catalyst at 110 °C for 2 h, was evaluated as shown in Table 1 and scheme 2. At first, the reaction was conducted using 1 mol% of the complex (precatalyst) with a molar ratio of *p*-bromoacetophenone (**5a**)/phenylboronic acid (**6a**)/TBAB/KOH: 1/1.2/0.6/2, to give 100% conversion of 4-acetyl-1,1′-biphenyl (**7a**) based on GC-analysis. In the second experiment, we used 0.75 mol% of the catalyst was used which gave full GC-conversion after 2 h at 110 °C. The reaction was repeated with different concentrations (mol%) of the catalyst **4** as shown in Table 1. In all cases, full conversion was obtained even in the presence of 0.001 mol% of the catalyst **4**. It can be concluded, from the data in Table 1, that the catalyst **4** showed excellent catalytic activity. Interestingly, the starting material was completely recovered unchanged when the reaction was carried out without the catalyst **4** (entry 9, Table 1). The structure of the obtained 4-acetylbiphenyl product was confirmed by elemental analyses as well as spectroscopic data (see “Experimental section”).

**Table 1:** Effect of concentration of catalyst **4** on the coupling of *p*-bromoacetophenone with phenylboronic acid in water under thermal conditions

Entry	Catalyst **4** (mol%)	GC-yield %^a,b^
1	1	100
2	0.75	100
3	0.50	100
4	0.25	100 (96)
5	0.125	100
6	0.05	100
7	0.025	100
8	0.005	87
9	0.00	0

^a^Conditions: *p*-Bromoacetophenone/ phenylboronic acid/ TBAB/ base/ water: 1/1.2/ 0.6/ 2 / 5 mL, under thermal heating at 100–110 °C for 2 h

^b^Conversions were based on GC-analysis and the values between parenthesis refer to the isolated yields

Here, Pd-complexe serve as “dormant species” [37] that is not participate in the real catalytic cycle but considered as a source of a catalytically active species of unknown nature. However, the Pd(0) species was reported most likely to be the true active catalysts [38]. Therefore, the catalyst **4** may serve here as a reservoir that is indirectly involved in the catalytic cycle but is a source of release of a considerable amount of colloidal Pd(0) which can show catalytic activity at low concentrations.

#### Effect of solvent and base on Suzuki coupling of *p*-bromoacetophenone (**5a**) with phenylboronic acid (**6a**) under thermal conditions

**Scheme 3:**
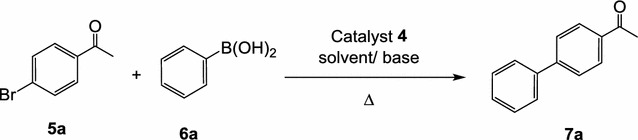
Base and solvent effects on the Suzuki coupling of p-bromoacetophenone (**5**) with phenylboronic acid (**6**)

In order to achieve efficient conversions and hence a maximum yield for the cross-coupling reaction, the various parameters and conditions that may affect such cross-coupling were optimized. Solvents and bases are among the most important controlling factors in such optimization. Actually, the selection of a base is still empirical, and no general rule for their choice has been used, therefore, the propriety of some bases and solvents for the coupling reaction between *p*-bromoacetophenone (**5a**) and phenylboronic acid (**6a**) were evaluated. As shown in Table 2 and scheme 3, in all cases, the catalyst **4** was used in 0.25 mol% concentration and the reaction was carried out thermally in different solvents, e.g. water, DMF, toluene and THF using potassium hydroxide or potassium carbonate as bases. The best result was obtained with water solvent in the presence of tetrabutylammonium bromide (TBAB) or cetyltributylammonium bromide (CTAB) as a co-catalyst after refluxing at 160 °C (entry 1 and 2, Table 2). The GC-conversion was 100% and the cross-coupled product 4-acetyl-1,1′-biphenyl (**7a**) was obtained in 96 and 92% isolated yield, respectively. Next, water was replaced with DMF, toluene and THF respectively, to give 80, 100 and 60% GC-conversions and in 51, 91 and 50% isolated yields, respectively. Next, replacement of KOH with K_2_CO_3_, as a base using water and DMF as solvents was also examined. Again, water proved itself as the good solvent compared with DMF (entry 3 and 5, Table 2).

**Table 2:** Base and solvent effects on the Suzuki coupling of *p*-bromoacetophenone (**5**) with phenylboronic acid (**6**) under thermal conditions

Entry	Base	Solvent	Yield %^a,b^
1	KOH	H_2_0 (TBAB)	100 (96)
2	KOH	H_2_0 (CTAB)	100 (92)
3	K_2_C0_3_	H_2_0 (TBAB)	100 (94)
4	KOH	DMF	80 (51)
5	K_2_C0_3_	DMF	80 (61)
6	KOH	Toluene	100 (91)
7	KOH	THF	60 (50)

^a^Conditions: *p*-Bromoacetophenone: 1 mmol; phenylboronic acid: 1.2 mmol; TBAB or CTAB: 0.6 mmol; base: 2 mmol; solvent: 5 mL, Pd-complex **4**: 0.25 mol%, heating for 2 h at 160 °C (H_2_O and DMF), 130 °C (Toluene) and at 90 °C (THF)

^b^Conversions were based on GC-analysis and the values between parenthesis refer to the isolated yields

The choice of solvent is decisive for Pd-catalysts, specifically its complexing properties. Non-aqueous solvents such as DMF can give supernatants which, unlike in cases of aqueous solvents, still show catalytic activity in C–C coupling reactions. Therefore, water as an eco-friendly and a green solvent and KOH as a cheap and common base are chosen for carrying out all the Suzuki–Miyaura cross-coupling reactions of aryl halides that are used in this work.

### Suzuki cross-coupling under microwave irradiation

**Scheme 4:**
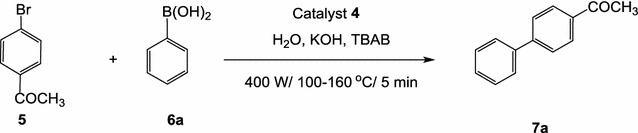
Suzuki cross-coupling of *p*-bromoacetophenone (**5**) with phenylboronic acid (**6**) under microwave irradiation

The model cross-coupling reaction in water using potassium hydroxide as a base and tetrabutylammonium bromide (TBAB), as a co-catalyst under microwave conditions at 100–160 °C for 5 min, was achieved as shown in Scheme 4. The reaction was conducted using 1 mol% of the catalyst **4** with a molar ratio of 4-bromoacetophenone (**5**)/phenylboronic acid (**6**)/TBAB/KOH: 1/1.2/0.6/2 to give 100% conversion and 96% isolated yield of 1,1′-biphenyl (**5**) based on TLC and ^1^H NMR analysis.

#### Suzuki coupling of aryl bromides with phenyl boronic acids using catalyst **4** under thermal heating and microwaves irradiation conditions

**Scheme 5:**
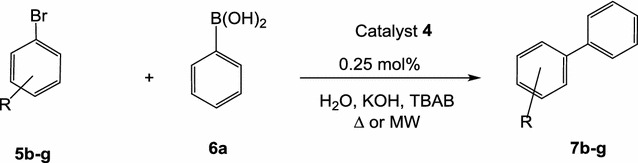
Suzuki coupling of aryl bromides **5b–g** with phenylboronic acid using the catalyst **4**

Applying the optimized conditions, Suzuki coupling between different aryl bromides **5b**–**g** and phenylboronic acid **6a**, under thermal heating conditions using the highly active catalyst **4**, afforded the corresponding biaryls in good yields (Scheme 5). Suzuki–Miyaura reaction of aryl bromides **5b**–**g** with the phenylboronic acid **6a** was performed using the catalytic system: water/KOH/TBAB, in the presence of 0.25 mol% of the catalyst **4**. As shown in Table 3. The obtained results reflect the reasonable activity of the catalyst **4** towards various aryl bromides **5b**–**g**.

**Table 3:** Suzuki coupling of aryl bromides **5b**–**g** with phenylboronic acid using the catalyst **4** under thermal  and microwave conditions

Entry	R	Conversion	∆ yield %	MW yield %
1	H	100	78	92
2	2-COCH_3_	100	61	93
3	4-OCH_3_	100	70	90
4	4-COOH	100	74	89
5	4-CH_3_	100	24	60
6	4-OH	100	77	87

Conditions: Bromide: 1 mmol; phenylboronic acid: 1.2 mmol; TBAB: 0.6 mmol; KOH: 2 mmol; water: 5 mL, Pd-complex **4**: 0.25 mol%, microwave heating (300 W) at 110 °C for 10 min and thermal heating at 100 °C for 3 h

### Suzuki cross-coupling reactions of other aryl halides

**Scheme 6:**
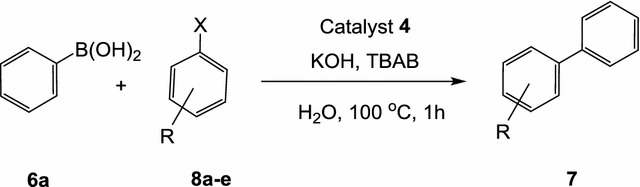
Suzuki coupling of aryl halides **8a–c** with phenylboronic acid

Next, the cross-coupling reaction between phenylboronic acid (**6**) and the haloaromatics **8a**–**c**, in water using potassium hydroxide as a base and tetrabutylammonium bromide (TBAB) as a co-catalyst under thermal conditions at 100 °C for 1 h, was evaluated as shown in Table 4 and scheme 6. At first, the reaction was conducted using 1 mol% of the catalyst **4** with a molar ratio of haloaromatics (**8**)/phenylboronic acid (**6**)/TBAB/KOH: 1/1.2/0.6/2 to give 100% conversion of 1,1′-biphenyl (**7b**) based on TLC and ^1^H NMR analysis. In all cases, full conversions were obtained as shown in Table 4, the catalyst **4** is efficient for the cross-coupling of **8** with **6** at the concentration 1 mol% catalyst.

**Table 4:** Suzuki coupling of aryl halides **8a**–**c** with phenylboronic acid using the catalyst **4** under thermal conditions

Entry	X	R	Yield %^a,b^
1	CI	H	100 (90)
2	CI	4-OH	90 (82)
3	CI	3-NH_2_	85 (74)
4	Br	H	100 (78)
5	|	H	100 (82)

^a^Conditions: haloaromatic/ boronic acid/ KOH/ TBAB /water (5 mL): 1/1.2/2/0.6, at 100 °C for 1 h

^b^Conversions were based on ^1^H NMR of the crude product and the values between parentheses refer to the isolated yields

### Suzuki cross-coupling reactions of halo heteroaromatics

**Scheme 7:**
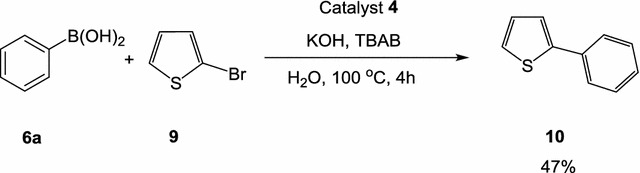
Suzuki cross-coupling reaction of 2-bromothiophene with phenylboronic acid using catalyst **4** under thermal conditions

The thiophene ring is a π-electron-rich heterocycle and consequently 2-bromothiophene (**9**) is considered as deactivated bromide in Pd-catalyzed C–C coupling reactions. Thus, the cross-coupling reaction between phenylboronic acid (**6**) and 2-bromothiophene (**9**), in water using potassium hydroxide as a base and tetrabutylammonium bromide (TBAB) as a co-catalyst, under thermal conditions at 100 °C for 1 h, was evaluated (Scheme 7). The reaction was conducted using, in each case, 1 mol% of the catalyst **4** with a molar ratio of 2-bromothiophene (**9**)/phenylboronic acid (**6**)/TBAB/KOH: 1/1.2/0.6/2. A full conversion of 2-phenylthiophene (**10**) was observed on the basis of TLC analysis (Scheme 4). Unfortunately, Coupling of 2-bromothiophene with phenylboronic acid in water, under thermal heating, was not efficient where poor yield was obtained and some unidentifiable byproducts were obtained.

#### Suzuki coupling of *p*-bromoacetophenone with arylboronic acids using complex **4** under thermal heating as well as microwave irradiation

**Scheme 8:**
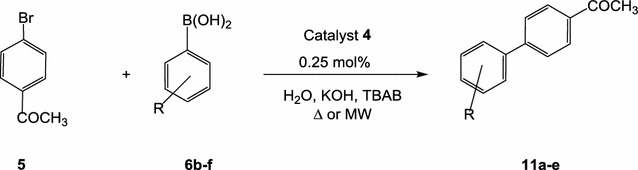
Suzuki coupling of p-bromoacetophenone (**5**) with arylboronic acids using the catalyst **4**

The optimized conditions using the highly active catalyst **4** was next applied in the Suzuki coupling between 4-bromoacetophenone (**5**) and arylboronic acids **6b**–**f**, under thermal heating as well as microwave irradiation conditions (Scheme 8). The Suzuki reaction of 4-bromoacetophenone (**5**) with the arylboronic acids **6b**–**f** was performed using the catalytic system; water/KOH/TBAB in the presence of 0.25 mol% of the catalyst **4** (Table 5). The obtained results reflect the high activity of the precatalyst **4**.

**Table 5:** Suzuki coupling of *p*-bromoacetophenone (**5**) with arylboronic acids using the catalyst **4** under thermal heating and microwave irradiation conditions

Entry	R	Conversion	∆ yield %	MW yield %
1	4-CH_3_	100	90	97
2	4-CI	100	93	94
3	4-F	100	82	90
4	3-NH_2_	100	76	96
5	2,4,6-(CH_3_)_3_	100	83	90

Conditions: Bromide: 1 mmol; arylboronic acid: 1.2 mmol; TBAB: 0.6 mmol; KOH: 2 mmol; water: 5 mL, Pd-complex: **4**: 0.25 mol%, microwave heating (400 W) at 160 °C and thermal heating at 100 °C

## Experimental section

### Materials and methods

All melting points were measured on a Gallenkamp melting point apparatus. The infrared spectra were recorded in potassium bromide discs on a Pye Unicam SP 3–300 and Shimadzu FT IR 8101 PC infrared spectrophotometers. The NMR spectra were recorded in deuterated chloroform (CDCl_3_) or dimethyl sulfoxide (DMSO-*d*_*6*_). On a Varian Mercury VXR-300 NMR spectrometer. Chemical shifts were related to that of the solvent. Mass spectra were recorded on a Shimadzu GCMS-QP1000 EX mass spectrometer at 70 eV. Elemental analyses were recorded on a Elementar-Vario EL automatic analyzer at the Micro-analytical Centre of Cairo University, Giza, Egypt. Formamidine **3** is prepared according to our pervious reported work [39] (Scheme 6). The Microwave irradiation was carried out on a CEM mars machine. CEM has several vessel types that are designed for their ovens: Closed-system vessels including the HP-500 (500 psig material design pressure and 260 °C), pictured below, have liners are composed of PFA and are ideal for many types of samples. HP-500 Plus vessels are ideal for routine digestion applications. Process up to 14 high-pressure vessels per run with temperatures up to 260 °C or pressures up to 500 psi (Scheme 7).

### Synthesis of the Pd(II)-complex (**4**)

A solution of sodium tetrachloropalladate (1 mmol), in methanol (2 mL) was added dropwise to a stirred solution of the formamidine **3** (1 mmol) in methanol (10 mL). After stirring for 1 h, the yellow precipitate was filtered off, washed with methanol and dried. The complex **4** was obtained as yellow powder (70%). mp 250 °C; 1H NMR (DMSO-*d*_*6*_) δ 356 (s, 6H, 2CH_3_), 6.53–6.55 (m, 2H, Py-H), 7.25–7.27 (d, 1H, Py-H), 7.46–7.48 (d, 1H, Py-H), 8.35 (s, 1H, CH); Anal. Calcd for C_16_H_14_Cl_2_N_2_OPdS: C, 41.80; H, 3.07; N, 6.09. Found: C, 41.68; H, 3.31; N, 6.03.

### Suzuki coupling of simple aryl halides

#### Effect of concentration of the Pd-complex **4** on the Suzuki coupling of 4-bromoacetophenone with phenylboronic acid in water under thermal conditions

A mixture of 4-bromoacetophenone (**5**) (199 mg, 1 mmol) and phenylboronic acid (**6a**) (146 mg, 1.2 mmol), tetrabutylammonium bromide (TBAB) (194 mg, 0.6 mmol), Pd-complex **4** (1 mol%), KOH (112 mg, 2 mmol) and water (10 mL) was stirred at 110 °C under open air for 2 h to give 4-acetyl-1,1′-biphenyl (**7**). The same experiment was repeated using Pd-complex **4** in 0.75 mol%. The amount (mol%) of the Pd-complex **4** was changed with respect to 4-bromoacetophenone (0.5, 0.25, 0.125, 0.05, 0.025, and 0.005 mol% of Pd-complex **4** with scales: 1, 1, 2, 5, 10, and 20 mmol of 4-bromoacetophenone, respectively). The molar ratio of the reaction components were, in all cases, as follows; 4-bromoacetophenone, phenylboronic acid, TBAB, KOH, water: 1/1.2/0.6/2/10 mL water (Scheme 8). The yield% versus concentration of Pd-complex **4** is outlined in Table 1.

*4*-*Acetyl*-*1,1′*-*biphenyl (* ***7a*** *)* White solid; mp. 118–120 °C (lit. mp. 119–120 °C); ^1^H NMR (CDCl_3_) δ 2.64 (s, 3H, CO CH_3_), 7.38–7.52 (m, 3H), 7.66–7.70 (d, 2H, *J* = 6.9 Hz), 7.71 (d, 2H, *J* = 7.5 Hz), 8.03 (d, 2H, *J* = 7.5 Hz); MS *m/z* (%) 196 (49.3, M^+^), 181 (100), 152 (61.4), 127 (5.2), 76 (9).

#### Effect of base and solvent on Suzuki cross-coupling of 4-bromoacetophenone with phenylboronic acid under thermal heating

A mixture of 4-bromoacetophenone (**5**) (199 mg, 1 mmol) and phenylboronic acid (**6a**) (146 mg, 1.2 mmol), TBAB (194 mg, 0.6 mmol) (in case of using water as a solvent), Pd-complex **4** (0.25 mol%), a base (2 mmol) and solvent (10 mL) was stirred under reflux in open air for 2 h to give acetyl-1,1′-biphenyl (**7**). The molar ratio of the reaction components were, in all cases, as follows; 4-bromoacetophenone, phenylboronic acid, tetrabutylammonium bromide (in case of water), base, solvent: 1/1.2/0.6/2/10 mL. The yield% versus different solvents and bases is outlined in Table 2.

#### Effect of base and solvent on Suzuki cross-coupling of 4-bromoacetophenone with phenylboronic acid under microwave heating

A mixture of 4-bromoacetophenone (**5**) (199 mg, 1 mmol) and phenylboronic acid (**6a**) (146 mg, 1.2 mmol), TBAB (194 mg, 0.6 mmol), Pd-complex **4** (0.25 mol%), KOH (112 mg, 2 mmol) and water (10 mL) was lunched in the specified CEM reaction vessel HP-500 at a given temperature for 5 min to give acetyl-1,1′-biphenyl (**7**).

### Suzuki cross-coupling of other aryl halides with phenylboronic acid in water under thermal heating

#### General procedure

A mixture of the appropriate aryl halides **5** or **8** (1 mmol), and phenylboronic acid (**6a**) (146 mg, 1.2 mmol), tetrabutylammonium bromide (194 mg, 0.6 mmol), Pd-complex **4** (0.25 mol%), KOH (112 mg, 2 mmol), and distilled water (5–10 mL) was stirred at 110 °C in open air until the reaction was complete (TLC-monitored) as listed in Tables 3 and 4. The cross-coupled product was then extracted with ethyl acetate (3 × 20 mL). The combined organic extracts were dried over anhydrous MgSO_4_ then filtered and the solvent was evaporated under reduced pressure. The residue was then subjected to separation via flash column chromatography with *n*-hexane/EtOAc (9:1) as an eluent to give the corresponding pure cross-coupled products **7b**–**g**.

### Suzuki cross-coupling of aryl bromides with phenylboronic acid in water under microwave irradiation

#### General procedure

A mixture of the appropriate aryl bromides **5** (1 mmol), and phenylboronic acid (**6a**) (146 mg, 1.2 mmol), tetrabutylammonium bromide (194 mg, 0.6 mmol), Pd-complex **4** (0.25 mol%), KOH (112 mg, 2 mmol), and distilled water (10 mL) were mixed in the specified CEM reaction vessel HP-500. The mixture was heated under microwave irradiating conditions at 110 °C and 300 Watt for 10 min. After the reaction was complete (monitored by TLC), the reaction mixture was extracted with ethyl acetate (3 × 20 mL). The combined organic extracts were dried over anhydrous MgSO_4_ then filtered and the solvent was evaporated under reduced pressure. The products **7b**–**g** were purified by flash column chromatography as described above. The yields% are outlined in Table 3.

*1,1′*-*Biphenyl (* ***7b*** *)* ^1^H NMR (CDCl_3_) δ 7.34–7.40 (m, 2H), 7.45-7.56 (m, 6H), 8.26 (d, 2H, *J* = 8.1 Hz); MS *m/z* (%) 154 (36.8, M^+^), 77 (100), 50 (42.1).

*2*-*Acetylbiphenyl (* ***7c*** *)* ^1^H NMR (400 MHz, CDCl3) δ: 7.57–7.49 (m, 4H); 7.45–7.38 (m, 3H); 7.37–7.33 (m, 2H); 2.01–1.99 (s, 3H); 13C NMR (100 MHz, CDCl3) δ: 205.0, 141.2, 140.9, 140.8, 130.9, 130.5, 129.1, 128.9, 128.1, 127.7, 30.6; MS: 196 (M^+^), 181, 152.

*4*-*Methoxy*-*1,1′*-*biphenyl (* ***7d*** *)* ^1^H NMR (CDCl_3_) δ 3.87 (s, 3H, –OCH_3_), 6.99 (d, 2H, *J* = 8.7 Hz), 7.31–7.45 (m, 3H), 7.54 (d, 2H, *J* = 9 Hz), 7.57 (d, 2H, *J* = 7.2 Hz); MS (*m/z*) (%) 184 (100, M^+^), 169 (54.0), 141 (37.4), 115 (16.6), 89 (12.5), 76 (49.8), 63 (25.7).

*4*-*phenylbenzoic acid (* ***7e*** *)* ^1^H NMR (500 MHz, DMSO-d6): δ (ppm) 13.17(s, 1H), 8.03 (d, J = 8.5 Hz, 2H), 7.79 (d, J = 8.0 Hz, 2H), 7.74 (t, J = 4.2 Hz, 2H), 7.51 (t, J = 7.5 Hz, 2H), 7.42 (t, J = 7.2 Hz, 1H); 13C NMR (125 MHz, DMSO-d6): δ (ppm) 167.4, 143.8, 139.1, 130.5, 129.9, 129.0, 128.2, 126.9, 126.6.

*4*-*Methylbiphenyl (* ***7f*** *)* ^1^H NMR (400 MHz, CDCl3) δ: 7.67–7.26 (m, 9H); 2.492.41 (m, 3H); 13C NMR (100 MHz, CDCl3) δ: 141.5, 138.7, 137.3, 129.8, 129.0, 127.3, 127.3, 21.4; MS: 168 (M^+^), 152.

*4*-*Hydroxy*-*1,1′*-*biphenyl (* ***7g*** *)* ^1^H NMR (CDCl_3_) δ 5.05 (s, 1H, OH), 6.92 (d, 2H, *J* = 7.8 Hz), 7.30–7.38 (m, 1H), 7.40–7.45 (m, 2H), 7.49 (d, 2H, *J* = 8.1 Hz), 7.56 (d, 2H, *J* = 8.4 Hz); MS m/z (%) 170 (100, M^+^), 141 (32.3), 115 (20.0), 63 (10.3), 51 (12.9).

### Suzuki coupling of 2-bromothiophene with phenylboronic acid in water under thermal conditions

A mixture of 2-bromothiophene **9** (1 mmol) and phenylboronic acid (**6a**) (1.2 mmol), tetrabutylammonium bromide (TBAB) (194 mg, 0.6 mmol), the Pd-complex **4** (1 mol%), KOH (112 mg, 2 mmol) in water (10 mL) was stirred at 110 °C in open air and the reaction was monitored by TLC. After the reaction was completed, the cross-coupling products were then extracted with ethyl acetate (3 × 20 mL). The combined organic extracts were dried over anhydrous MgSO_4_ then filtered and the solvent was evaporated under reduced pressure. The residue was then subjected to a flash column chromatography with *n*-hexane/EtOAc (10:1) as an eluent to give the corresponding pure 2-phenylthiophene **10**.

*2*-*Phenylthiophene (* ***10*** *)* ^1^H NMR (CDCl_3_) δ 7.02 (d, 1H, *J* = 3.0 Hz), 7.06 (d, 1H, *J* = 3.6 Hz), 7.08–7.17 (m, 3H), 7.33–7.40 (m, 2H), 7.49 (d, 1H, *J* = 7.8 Hz), 7.59 (d, 1H, *J* = 7.8 Hz); MS *m/z* (%) 160 (M^+^, 100), 134 (33.8), 115 (56.1), 102 (14. 7), 63 (35.5), 45 (56.2).

### Suzuki coupling of 4-bromoacetophenone with arylboronic acids in water under microwave irradiation condition

A mixture of 4-bromoacetophenone (**5**) (1 mmol) and the appropriate arylboronic acid **6** (1.2 mmol), tetrabutylammonium bromide (TBAB) (194 mg, 0.6 mmol), the Pd-complex **4** (0.25 mol%), KOH (112 mg, 2 mmol) in water (10 mL) was refluxed (under thermal conditions) or mixed in a process glass vial (under microwave irradiation conditions). After the reaction was complete, the cross-coupled products were then extracted with EtOAc (3 × 20 mL). The combined organic extracts were dried over anhydrous MgSO_4_ then filtered and the solvent was evaporated under reduced pressure. The residue was then subjected to separation via flash column chromatography with *n*-hexane/EtOAc (10:1) as an eluent to give the corresponding pure cross-coupled products **11a**–**e** (Table 5).

*4*-*Acetyl*-*4′*-*Methy*-*1,1′*-*biphenyl (* ***11a***) ^1^H NMR (CDCl_3_) δ 2.42 (s, 3H, Ar CH_3_), 2.64 (s, 3H, CO CH_3_), 7.26 (d, 2H), 7.53 (d, 2H), 7.68 (d, 2H), 8.03 (d, 2H); MS *m/z* (%)210 (70.9, M^+^).

*4*-*Acetyl*-*4′*-*Chloro*-*1,1′*-*biphenyl (* ***11b***) ^1^H NMR (CDCl_3_) δ 2.64 (s, 3H, CO CH_3_), 7.33(d, 2H), 7.63 (d, 2H), 7.76 (d, 2H), 8.02 (d, 2H); MS *m/z* (%) 230 (59, M^+^).

*4*-*Acetyl*-*4′*-*fluoro*-*1,1′*-*biphenyl (* ***11c*** *)* ^1^H NMR (CDCl_3_) δ 2.64 (s, 3H, CO CH_3_), 7.14–7.16 (m, 2H), 7.57–7.65 (m, 4H), 8.202 (d, 2H); MS *m/z* (%) 214 (47, M^+^).

*4*-*Acetyl*-*3′*-*amino*-*1,1′*-*biphenyl (* ***11d*** *)* ^1^H NMR (CDCl_3_) δ 2.63 (s, 3H, CO CH_3_), 3.74 (br, 2H), 6.73 (d, 1H), 6.93 (s, 1H), 7.00–7.03 (1, 2H), 7.25–7.28 (t, 1H), 7.66 (d, 2H), 8.01 (d, 2H); MS *m/z* (%) 211 (64, M^+^).

*4*-*Acetyl*-*2′,4′,6′*-*trimethyl*-*1,1′*-*biphenyl (* ***11e*** *)* ^1^H NMR (CDCl_3_) δ 2.01 (s, 6H, 2 Ar–CH_3_), 2.53 (s, 3H, Ar–CH_3_), 2.66 (s, 3H, CO–CH_3_), 6.97 (s, 2H), 7.28 (d, 2H), 8.05 (d, 2H); MS *m/z* (%) 238 (31.6, M^+^).

## Conclusions

In conclusion, we developed a new and an efficient Pd-complex catalyst for Suzuki C–C cross-coupling of aryl halides with aryl boronic acids under green methodology. The activity of the pyridylformamidine based Pd-complex is high even at low mol% concentrations in the Suzuki cross-coupling between aryl bromides and arylboronic acids in water under microwave irradiation.
